# The Week's Work

**Published:** 1911-07-15

**Authors:** 


### The Week's Work.

### THE KING AND THE DUBLIN HOSPITALS.

A telegram from Dublin Castle states that: "The King has received from Lord Iveagh the munificent gift of £50,000, which he asks may be distributed at his Majesty's pleasure to hospitals in Dublin, together with the Consumption Hospital at Newcastle, county Wicklow." We learn that the following gentlemen have been asked to serve on a committee to advise his Majesty as to the best manner in which to give effect to Lord Iveagh's gift: The Lord O'Brien, the Right Hon. Jonathan Hogg, L. A. Waldron, Esq., Joseph Todhunter Pim, Esq., and Colonel Addison. The decision of this committee will be awaited with the greatest interest, and his Majesty's superintendence over its deliberation will make his attention to the Irish Hospitals probably the most remembered incident of his present visit.

### MR. PASSMORE EDWARDS' WILL.

Mr. John Passmore Edwards, formerly editor and proprietor of the *Echo*, and M.P. for Salisbury, an account of whose numerous hospital foundations was given in The Hospital of April 29 (page 121), has left estate of the gross value of £47,685, of which the net personalty has been sworn at £47,411. It is interesting to observe that Mr. Passmore Edwards by his will made no bequests for public uses.

### A GREAT DAY FOR THE SALFORD ROYAL HOSPITAL.

On Thursday, July 6, the efforts which were decided upon in June 1906 were completed at the Salford Royal Hospital by the opening of the King Edward VII. Memorial Wing, the nurses' home, the new wards, the theatre, and out-patients' department, in the presence of Lord Shuttleworth, the Lord-Lieutenant of Lancashire. We congratulate the hospital in the persons of Mr. Lawrence Pilkington, chairman of the board of management, and Mr. George Ruddle, the secretary, not merely on what is virtually an entirely new institution, but also on the capitally-prepared and illustrated guide to the new buildings, which has been issued in the form of a separate report. This will form one of the important chapters in the future history of the hospital. The story of these five years of work is attractively told, and the plans which accompany the text make the progressive enlargements easily understandable by even the most casual of subscribers. The opening ceremony and the speeches we report elsewhere, adding here only a rough summary of the vast additions to the original building and site. On the east of the hospital an important piece of land was obtained for £11,250, and Mr. Keith Young was appointed assessor. The architect finally appointed was Mr. John Ely, F.R.I.B.A. In June 1907 an appeal for £70,000 was issued, one-third of which was promptly subscribed. Mr. Edward Graham Wood, the chairman of the Extension Committee, indeed, was able to announce in October last that £50,000 had been raised. This fund was sufficient to pay for the buildings, which consist of the memorial wing, with its 45 beds, of the 71 nurses' bedrooms, with 11 bathrooms, and which comprise a total of 209 beds. Approximately, £14,000 has yet to be raised, and we hope that Sir William Mather, the president, will see this done at an early date.

### THE CHAIRMANSHIP OF THE COVENTRY HOSPITAL

We join in the hope that is felt by the institutional staff of the Coventry and Warwickshire Hospital that Mr. Alfred Herbert, the Chairman of the Board of Management, may be induced to postpone his resignation until the extensions are completed, or at least until the foundation-stone of the King Edward Wing has been laid. This would be a very graceful and a very helpful conclusion to his work for the hospital, and is a conclusion, moreover, that we have little doubt Mr. Herbert may be induced to accept.

### CONSUMPTION DISPENSARIES FOR LONDON?

Lord Glenconner and Sir Alexander Henderson have issued an appeal on behalf of a central fund for establishing in the London districts local dispensaries for incipient phthisic cases. They state that £26,000 has been raised towards the £100,000 for which they appeal, and support their claim by reference to the dispensaries already at work in Bermondsey, Stepney, Marylebone, N. Kensington, and Paddington, and to the adoption of similar dispensaries in Canada and on the Continent. The care and attention, apart from anything else, by which these dispensaries tend to replace the carelessness and neglect that prevail in the homes of the poor are almost certain to produce good results, and it will be interesting to see exactly how far the report of the Royal Commission on Tuberculosis brings any fresh evidence to show the lines on which such dispensaries have proved most useful. In so far as these dispensaries tend to attract to them incipient cases and to inculcate a few maxims as to the more obvious ways of avoiding infection, they will assist and relieve the work with which hospital out-patient departments at present are over-run or at best ineffective.

### A NOVEL EXPERIMENT AT PADDINGTON.

An enterprise of considerable interest was inaugurated on Thursday last week by the opening of Kensal House School for Tuberculous Children at 535 Harrow Road, in the presence of the Duke of Argyll. Mr. Edward White, J.P., the Chairman of the London County Council, reminded his hearers that the experiment was the joint undertaking of the Paddington and Kensington Dispensary for the prevention of consumption and of the London County Council. The Dispensary was started in 1909, and the success of its work has led it to suggest the education, under special conditions, of the phthisic children in its two boroughs.

The London County Council embraced the proposal and has undertaken to supply the teaching staff, while the Dispensary Committee, with the aid of £1,750 from the Ogilvie Trust, has purchased the site, or (for the speakers were indistinctly heard in the open air) leased the house and garden for three years. The new school is, we believe, the first of its kind, and the 110 children who will enjoy its advantages of open-air education are provided, for London, with a large garden, and with the well-ventilated class-rooms that a converted private house is able to give.

### SOME POINTS OF INTEREST IN THE DISPENSARY SCHOOL.

The experiment has many points of interest. It is a striking instance, as the lady chairman of the Paddington Board of Guardians pointed out in a very capable speech, of the co-operation which has now become possible between the County Council and local bodies. It offers a chance of extending a system of devolution, which will give the managers of the school a free hand, and which might relieve the County Council of its present absurdly multiplied detail work. It opens up also a vista of problems. For instance, as Dr. R. Dudfield, the chairman of the Dispensary Committee, remarked, the experiment, if it prove a success, may lead to the day-school system being abandoned for tuberculous children. Their teachers, for instance, must feel how much their work will suffer from the substitution of an inferior environment when the child returns home at night. Sanatoria schools might be the outcome. Further than this, what arrangements will be made for feeding these tuberculous children? Hospital managers would certainly suggest the provision of their meals at the school. Is the hygienic advantage of this common-sense course to be abandoned because the feeding of school children is now a party question? It remains to be seen what Miss A. M. Hutchinson, the headmistress, will be able to accomplish under existing conditions. Hospital workers, any way, will welcome this experiment as an organised attempt to deal with a class of patient for which their own out-patient departments are practically useless, though they are kept busy tinkering at cases which the present school is capable of arresting under the special conditions which it provides.

### A NOTE FOR HOSPITAL DISPENSERS.

By an Order in Council, preparations containing more than five per cent. by weight of free ammonia must be sold in bottles distinguishable by touch from ordinary bottles, and which must be labelled with the word "Poisonous" and the words "Not to be taken" in addition to the name and address of the seller. The order is made under Section 5 of the Poisons and Pharmacy Act, 1908, and takes effect on February 1, next year. Like the mineral acids to which the above section was made applicable by an Order made earlier in the year, ammonia is responsible for a large number of accidental deaths and of cases of suicide. There is ground for hope that when the sale of ammonia is subject to these restrictions the annual death rate from ammonia poisoning will be diminished, and the work of hospital dispensers made more easy. The system of having bottles of authorised shapes for defining their dangerous contents is one that has been in force in German Hospitals for some years.

### SEAL-STAMPS FOR HOSPITALS?

The acknowledged difficulty in raising the income of the voluntary hospitals, albeit that its total is increasing, makes it worth while for hospital secretaries to consider any new proposition for raising money that is respectable and legal. We present therefore a scheme that its supporters allege to be a very popular and successful means of raising money on the Continent. This scheme is one for the issue on behalf of the hospitals of seal-stamps, which would bear on their face an artistic design and a taking motto, and which are affixed like wafers to backs of envelopes. The supporters of a hospital purchase these in Germany from the post offices and the tobacconists, and the price, which varies from one penny to a shilling, goes to the charity, which is also advertised every time a wafer-stamped letter goes through the post. The stamps are not labels, for they do not assist the package in arriving at its destination; they are wafers or seals, and the Governments, of Sweden and Denmark in particular, receive a percentage on the sales. The question of their distribution is made harder in this country, we understand, from the refusal of our Post Office to assist the scheme, and the Union of Philanthropic Philately has been formed to test public opinion in the matter. Its secretary is Mr. John S. Madden, care of Messrs. Dunkerton, solicitors, 23 Bedford Row, W.C., who will give detailed answers to any questions addressed to him. It is worth remembering that the scepticism which has led many to ask who will trouble to purchase these stamps, or to affix them to the back of letters when purchased, is answered by the success of the stamps in certain countries abroad. We may recall also the success which the Crystal Palace is gaining at the moment from the stamp it has issued, and the adoption by Sir William Treloar's Home of a similar scheme. It would be interesting to have our readers' opinions as to the feasibility of its adoption for hospitals in this country. Has the London, as one of our most diversely advertised hospitals, ever considered the merits of an issue of stamps? Some were issued in connection with the Prince of Wales's Hospital Fund in 1897, and realised, we believe, over £30,000.

### THIS WEEK'S DRUG MARKET.

Business in the drug market has been quieter again during the week, but several articles have continued to attract attention. Opium is not quotably higher in price, but the strong position is well maintained and a further advance is considered probable; the same remarks apply to morphine and codeine. Mercury has been slightly advanced in price. Menthol is tending dearer. Oil of cloves is slightly dearer, as also is carbolic acid. Oil of aniseed and essence of lemon are tending rather higher in price.

